# Linking the Developmental and Degenerative Theories of Schizophrenia: Association Between Infant Development and Adult Cognitive Decline

**DOI:** 10.1093/schbul/sbu010

**Published:** 2014-03-01

**Authors:** Hiroyuki Kobayashi, Matti Isohanni, Erika Jääskeläinen, Jouko Miettunen, Juha Veijola, Marianne Haapea, Marjo-Riitta Järvelin, Peter B. Jones, Graham K. Murray

**Affiliations:** ^1^Department of Psychiatry, University of Cambridge, Cambridge, UK;; ^2^Department of Neuropsychiatry, School of Medicine, Toho University, Tokyo, Japan;; ^3^Department of Psychiatry, University of Oulu and Oulu University Hospital, Oulu, Finland;; ^4^Department of Epidemiology and Biostatistics, MRC-HPA Centre for Environment and Health, Imperial College London, London, UK;; ^5^Institute of Health Sciences, University of Oulu, Oulu, Finland;; ^6^Biocenter Oulu, Oulu, Finland;; ^7^Department of Life Course and Services, National Institute for Health and Welfare, Oulu, Finland;; ^8^Behavioural and Clinical Neuroscience Institute, University of Cambridge, Cambridge, UK

**Keywords:** schizophrenia, cognition, executive function, memory, neurodevelopment

## Abstract

Neurodevelopmental and neurodegenerative theories may be viewed as incompatible accounts that compete to explain the pathogenesis of schizophrenia. However, it is possible that neurodevelopmental and neurodegenerative processes could both reflect common underlying causal mechanisms. We hypothesized that cognitive dysfunction would gradually deteriorate over time in schizophrenia and the degree of this deterioration in adulthood would be predicted by an infant measure of neurodevelopment. We aimed to examine the association between age of learning to stand in infancy and deterioration of cognitive function in adulthood. Participants were nonpsychotic control subjects (*n* = 76) and participants with schizophrenia (*n* = 36) drawn from the Northern Finland 1966 Birth Cohort study. The schizophrenia group showed greater deterioration in abstraction with memory than controls, but there were no differences between schizophrenia and controls in rate of change of other cognitive measures. Age of learning to stand in infancy significantly inversely predicted later deterioration of abstraction with memory in adult schizophrenia (later infant development linked to greater subsequent cognitive deterioration during adulthood), possibly suggesting a link between abnormal neurodevelopmental and neurodegenerative processes in schizophrenia.

## Introduction

Several general population-based cohort studies have demonstrated significant associations between delay of infant motor development and the later onset of schizophrenia.^1–4^ Delayed motor development may reflect aberrant functional maturation of cortical-subcortical circuits, as motor delay predicts not only behavioral problems in later childhood and adolescence^5^ but also development of higher cognitive function, educational achievements, and brain structure in adulthood.^6–8^ Consistent with this line of research, longitudinal studies have also revealed that individuals who subsequently developed schizophrenia manifested cognitive deficits, including executive dysfunction, before the onset of the illness,^1,9–11^ suggesting that abnormal neurodevelopment is a critical part of the pathogenesis of schizophrenia.^12,13^

Another conceptualization of schizophrenia is that it is a disorder with a significant neurodegenerative component.^14^ Although neuropathological studies document intact numbers of neurons in schizophrenia with no evidence of gliosis,^15^ longitudinal studies have found progressive reduction in brain volume over time in patients with schizophrenia, suggesting that neurodegenerative processes may be ongoing throughout the course of the illness.^14,16–19^

It is possible that abnormal developmental processes may be responsible for some aspects of patients’ conditions, and (different) degenerative processes for other aspects. An alternative, more parsimonious, theory is that the same causal factors underpin both abnormal developmental processes and degenerative processes in schizophrenia.^20^ For example, the same genetic, in utero, or perinatal factors that cause abnormal neurodevelopment in schizophrenia could, conceivably, also cause increased degenerative processes later in the course of illness.^21^ Given that the pathogenetic mechanisms of schizophrenia are unknown, testing the theory that developmental and degenerative aspects of the disorder are related is challenging, but, we argue, not impossible. In this article, we provide evidence in favor of an account linking altered development and degeneration in schizophrenia. Here, we examine associations between proxy markers of abnormal neurodevelopment and neurodegeneration in schizophrenia, and test whether people with schizophrenia who are later in learning to stand in infancy also have the most pronounced decline over time in adult cognitive function.

## Methods

### Subjects

Control subjects and participants with schizophrenia were drawn from the Northern Finland 1966 Birth Cohort study, which is based on 12 068 pregnant women and their 12 058 children born alive in the provinces of Lapland and Oulu, with an expected delivery date during 1966. The live births in this study represent 96% of all births in the region.^22^ The Faculty of Medicine Ethics Committee of the University of Oulu continuously reviews the design of the Northern Finland 1966 Birth Cohort study.

### Age of Learning to Stand in Infancy

Developmental data on the age (in months) of the infant when he/she was first able to stand without support were prospectively collected during children’s visits to child welfare centers during childhood (the visits were frequent, with the average cohort member making 10 such visits in the first 12 mo of life). These routinely collected data were supplemented with developmental information obtained with a research examination of infant development performed by specialist nurses at the age of approximately 1 year. This information was collected at an age of 11.5 months or later from 96% cases in the cohort.^23^ From all available information, we extracted a continuous measure of age at learning to stand without support. We focused on this measure because of the temporal proximity of its attainment to the timing of the 1-year assessment, potentially increasing the reliability of the measure, and because our previous work has shown it to be a sensitive predictor of adolescent or adult cognitive, academic, and psychiatric outcomes.^2,24,25^

### First Cognitive Assessment (1999–2001, T1)

A detailed description and additional information regarding the cognitive assessment conducted in 1999–2001 (age 33), including analyses of the degree of selection bias, is available from previous reports.^6,26,27^ All members of this birth cohort who had developed schizophrenia (based on the Finnish Hospital Discharge Register)^28,29^ were invited for this assessment, which included examinations via a structured interview (Structural Clinical Interview for DSM-III-R; SCID) and neuropsychological testing. The subjects were rated according to the Positive and Negative Syndrome Scale, Clinical Global Impressions scale, and the Social and Occupational Functioning Assessment Scale (SOFAS) based on the SCID-I interview. General population control subjects were selected using gender-stratified random sampling: 187 controls were randomly selected to be invited. Sixty-one subjects who met the DSM-III-R criteria for schizophrenia and 104 controls attended the first cognitive assessment. One control subsequently developed psychosis and was excluded from the study analysis. All participants gave written informed consent prior to participation.

### Second Cognitive Assessment (2008–2010, T2)

Thirty-six subjects with schizophrenia and 76 nonpsychotic controls who undertook the first cognitive assessment completed the study by attending a second cognitive assessment 9 years later using the same measures.

The amount of antipsychotic medication exposure between T1 and T2 was calculated through the methods of dose-years. A dose-year is defined as the form of (chlorpromazine equivalent in milligram) × (time on dose measured in years), and is equivalent to the time in years on a dose equivalent to 100 mg chlorpromazine daily.^30^

### Measure of Abstraction and Measure of Abstraction With Memory

Executive function was assessed using the Abstraction, Inhibition, and Working Memory (AIM) task,^31^ which we have previously shown to be associated with adult brain structure in a fronto-striatal-cerebellar network and infant development in the general population.^6,7^ The AIM task is a computerized rule-abstraction/category-learning task that requires subjects to use information to group stimuli in a meaningful way on the basis of feedback received during the test. In this task, manipulation of information is operationalized as visual abstraction. Participants are shown 5 shapes: 2 shapes in the upper-right corner and 2 shapes in the upper-left corner of a computer screen. A fifth target object appears in the center of the screen below the other stimuli. The participant’s task is to pair the target object with the objects on either the left or right. In some trials, an additional maintenance requirement was superimposed on this basic module by adding a delay between the presentation of the target and the other objects. Thus, this task yields 2 outcome measures: total score on the abstraction trials and total score on the trials involving abstraction with memory.

### Visual Object Learning and Memory

Participants are shown a set of 10 visual objects—the learning set. In a forced choice paradigm, they are then are required to recognize those stimuli within a group of 20 objects, of which 10 are distractors. There are 4 trials, each with novel distractors; after each trial, the learning set is presented. The dependent variable is the total number of correct responses in the 4 trials summed.^32^

### Verbal Learning

The California Verbal Learning Test is a paper-and-pencil auditory verbal memory test using a 16-item shopping list (the learning set) that is read to the subject 5 times.^33^ After each trial, subjects must repeat back as many items as they can remember. The dependent variable is the overall score, achieved by summing the results of the 5 trials.

### Statistical Analyses

We compared demographic and clinical variables at baseline between group (schizophrenia and controls) using independent *t* tests for continuous variables and the chi-square test for categorical variables. *T* tests were used to compare change in cognitive function over time between groups.

In order to determine whether our study was affected by attrition bias, we examined whether subjects who participated in the cognitive examinations at both time points were representative of subjects who dropped out of follow-up by comparing the study completers and dropouts on a range of cognitive, developmental, clinical, and demographic variables, and where available, we used register data to compare people with schizophrenia who completed the study vs all cohort members with schizophrenia who did not complete the study, irrespective of whether or not they ever attended the study.

We planned that for any cognitive variable in which patients’ scores changed significantly differently to controls, we would then examine the association (Pearson’s *r*) between the degree of change in cognitive function during midlife and the age of learning to stand during infancy within groups with correlation tests. In the event, only one cognitive variable met this criterion (see “Results” section). We confirmed the correlation analyses with multiple linear regressions within groups using cognitive change as an outcome variable and age at learning to stand as a predictor, adjusting, in schizophrenia, for gender, education level, exposure to antipsychotic medication (dose-years), and social functioning (the SOFAS at T1 and T2). For the cognitive variable that showed a significant association with infant development in schizophrenia in a within group analysis, we then tested whether the strength of the association between infant development and change in adult cognition differed significantly in cases and controls by using an interaction analysis as follows: We pooled all subjects, used cognitive change as an outcome variable, and diagnosis, development, and (diagnosis × development) as predictors in a multiple linear regression.

## Results

### Demographic and Clinical Characteristics at Time 1 (T1)

The demographic characteristics of the current sample are described in table 1.

**Table 1.** Demographic and Clinical Characteristics at the Time of the First Cognitive Assessment

	Schizophrenia	Controls	Comparison
*N*	36	76	
Male; *n* (%)	21 (58)	46 (61)	χ^2^ = 0.05, *df* = 1, *P* = .84
Education			χ^2^ = 19.5, *df* = 2, *P* < .01
Basic (9 y or less); *n* (%)	7 (19)	3 (3.9)	
Secondary (10–12 y); *n* (%)	29 (81)	48 (63)	
Tertiary (over 12 y); *n* (%)	0 (0)	25 (33)	
Social class (parental)			χ^2^ = 1.46, *df* = 3, *P* = .71
Highest; *n* (%)	13 (36)	21 (28)	
Middle; *n* (%)	12 (33)	32 (42)	
Lowest; *n* (%)	7 (19)	12 (16)	
Farmers; *n* (%)	4 (11)	11 (14)	
PANSS total; mean (SD)	56.3 (19.0)	—	
CGI; mean (SD)	5.0 (1.2)	1.1 (0.5)	*t* = 18.82, *df* = 40.3, *P* < .01
SOFAS; mean (SD)	47.3 (14.6)	85.8 (5.3)	*t* = 15.34, *df* = 39.5, *P* < .01
Age of learning to stand without support (months); mean (SD)	11.2 (1.3); *n* = 29	10.3 (1.4); *n* = 62	*t* = 2.96, *df* = 89, *P* < .01

*Note*: CGI, Clinical Global Impression; PANSS, Positive and Negative Syndrome Scale; SOFAS, Social and Occupational Functioning Assessment Scale.

### Comparison Between Study Completers and Study Dropouts

Comparisons between participants who took part in both cognitive examinations and those who dropped out of the study after the first cognitive examination are presented in table 2. There was no difference between study completers and study dropouts in educational, sociodemographic, cognitive, developmental, or clinical measures, with the exception that control completers had a slightly higher baseline SOFAS score than control dropouts.

**Table 2.** Attrition Analysis of Cohort Members Who Participated in the First, But Not the Second, Cognitive Examination

	Schizophrenia (*N* = 58)		Statistics	Controls (*N* = 103)		Statistics
	Followed	Not Followed		Followed	Not Followed	
*N* (unless otherwise specified)	36	22		76	27	
Gender, male; *n* (%)	21 (58)	13 (59)	χ^2^ < 0.01, *P* > .99	46 (61)	16 (59)	χ^2^ = 0.01, *P* > .99
Secondary education; *n* (%)	29 (81)	18 (82)	χ^2^ = 0.01, *P* > .99	73 (96)	25 (93)	χ^2^ = 0.52, *P* = .60
Unemployed or retired; *n* (%)	21 (58)	12 (55)	χ^2^ = 0.08, *P* = .78	4 (5)	4 (15)	χ^2^ = 2.54, *P* = .20
Age of onset (years); mean (SD)	23.1 (4.4)	24.7 (3.4)	*t* = 1.46, *P* = .15	—	—	—
Current psychiatric medication at T1; *n* (%)	27 (75)	14 (67)	χ^2^ = 0.46, *P* = .55	1 (1)	0 (0)	χ^2^ = 0.36, *P* > .99
Current hospital care at T1; *n* (%)	2 (6)	1 (5)	χ^2^ = 0.02, *P* > .99	0 (0)	0 (0)	χ^2^ = 0.24, *P* = .44
CGI at T1; mean (SD)	5.0 (1.2)	4.6 (1.1)	*t* = −1.55, *P* = .13	1.1 (0.5)	1.2 (0.8)	*t* = 0.67, *P* = .51
SOFAS at T1; mean (SD)	47.3 (14.6)	50.1 (9.2)	*t* = 0.92, *P* = .36	85.8 (5.3)	82.3 (10.7)	***t* = 2.19**, ***P* = .03**
PANSS total at T1; mean (SD)	56.3 (19.0)	50.1 (16.2)	*t* = −1.26, *P* = .22	—	—	—
Age of learning to stand without support (months); mean (SD)	11.2 (1.3); *n* = 29	11.3 (0.7); *n* = 16	*t* = 0.46, *P* = .65	10.3 (1.4); *n* = 62	10.6 (1.3); *n* = 21	*t* = 0.88, *P* = .38
Abstraction at T1; mean (SD)	21.9 (4.2); *n* = 36	22.1 (5.3); *n* = 21	*t* = 0.16, *P* = .88	24.0(2.8); *n* = 73	24.3 (3.0); *n* = 27	*t* = 0.49, *P* = .63
Abstraction with memory at T1; mean (SD)	19.8 (4.4); *n* = 36	20.2 (5.0); *n* = 21	*t* = 0.35, *P* = .73	23.4 (3.7); *n* = 73	23.5 (3.2); *n* = 27	*t* = −0.20, *P* = .84
Verbal learning at T1; mean (SD)	46.4 (13.9); *n* = 35	45.3 (14.3); *n* = 22	*t* = 0.28 *P* = .78	59.8 (7.3); *n* = 74	60.0 (8.5); *n* = 26	*t* = 0.13, *P* = .89
Visual learning at T1; mean (SD)	58.5 (8.1); *n* = 33	61.1 (6.6); *n* = 20	*t* = 1.1, *P* = .26	68.7 (5.4); *n* = 75	68.4 (4.8); *n* = 26	*t* = 0.19, *P* = .85

*Note*: Abbreviations are explained in the first footnote to table 1.

### Comparisons in Register Data Between Schizophrenia Patients Who Were Study Completers and All Other Birth Cohort Members With Schizophrenia

There are 2 ways that birth cohort members could not complete the study: either through taking part in the first cognitive examination and dropping out, or through not taking part in the study at all (because they declined, or could not be traced, or had died before the study started). Analysis of register data allowed us to examine how representative our study completers were of all cohort members with schizophrenia, including patients whose death preceded the study. There was no significant difference between schizophrenia participants who completed the study and were included in the final analysis vs those cohort schizophrenia participants who did not complete the study (for any reason, including not starting the study) in the following variables: in age of learning to stand (mean [SD] 11.1 mo [1.3] in completers; 10.7 [1.4] in others; *t* = 1.1, *df* = 80, *P* = .27), in age of illness onset (mean [SD] completers 23.1 [4.4] vs others 22.5 [4.1], *t* = 0.62, *df* = 109, *P* = .54), in gender (completion rates: males 29%, females 39%, χ^2^ = 1.0, *P* = .40), in educational level (completion rate in patients with basic education 21%; with higher education 38%; χ^2^ = 3.14, *P* = .08).

### Cross-sectional Cognitive Function

Cognitive function at T1 and T2 for people with repeat cognitive measures is shown in table 3.

**Table 3.** Cognition in Participants Who Undertook Repeat Cognitive Function at Time 1 (Age 33–35) and Time 2 (9 y Later)

	Schizophrenia	*N*	Controls	*N*
Abstraction time 1; mean (SD)	21.8 (4.2)	34	23.9 (2.8)	68
Abstraction time 2; mean (SD)	22.0 (4.1)	34	24.6 (2.4)	68
Abstraction with memory time 1; mean (SD)	19.7 (4.5)	34	23.2 (3.7)	68
Abstraction with memory time 2; mean (SD)	17.5 (6.0)	34	24.0 (2.9)	68
Verbal learning time 1; mean (SD)	46.4 (14.1)	34	60.1 (6.8)	73
Verbal learning time 2; mean (SD)	43.5 (14.9)	34	55.1 (8.4)	73
Visual learning time 1; mean (SD)	58.3 (8.3)	31	68.6 (5.6)	68
Visual learning time 2; mean (SD)	58.6 (11.0)	31	68.6 (5.4)	68

*Note*: Mean cognitive function in was lower in schizophrenia than in controls (*P* < .005) in both time points in every test. Please note that numbers in table 3 may slightly differ from those in table 2, as not all participants completed all cognitive tests.

### Longitudinal Changes in Cognition (T2 − T1)

Cognitive difference scores were calculated by subtracting T1 performance from T2 performance, with a greater positive score indicating improvement (table 4). Whereas verbal learning, visual learning, and abstraction (without memory) were found to stay constant, or decline in accordance with normal ageing, a significant deterioration of abstraction with memory was observed in the schizophrenia group (−2.2 ± 5.5) compared to controls (0.8±3.5; *t* = 3.3, *df* = 100, *P* = .001). This deterioration remained significant after adjustment for level of education (*F* = 12.1, *df* = 1,99, *P* = .001) and for correction for multiple comparisons (*P* < .005 Bonferonni corrected).

**Table 4.** Cognitive Change Over Time in Schizophrenia Compared to Controls

	Schizophrenia	*N*	Controls	*N*	*T*	*P*
Change in abstraction	0.24 (4.2)	34	0.78 (2.8)	68	0.8	.44
Change in abstraction with memory	−2.2 (5.5)	34	0.80 (3.5)	68	3.3	.001*
Change in verbal learning	−2.8 (13.5)	34	−5.0 (6.8)	73	1.1	.27
Change in visual learning	0.32 (8.6)	31	0.01 (4.9)	68	0.2	.8

*P < .005 Bonferonni corrected.

### Association Between Age of Learning to Stand and Longitudinal Change in Abstraction With Memory in Schizophrenia and Controls

**Fig. 1.**
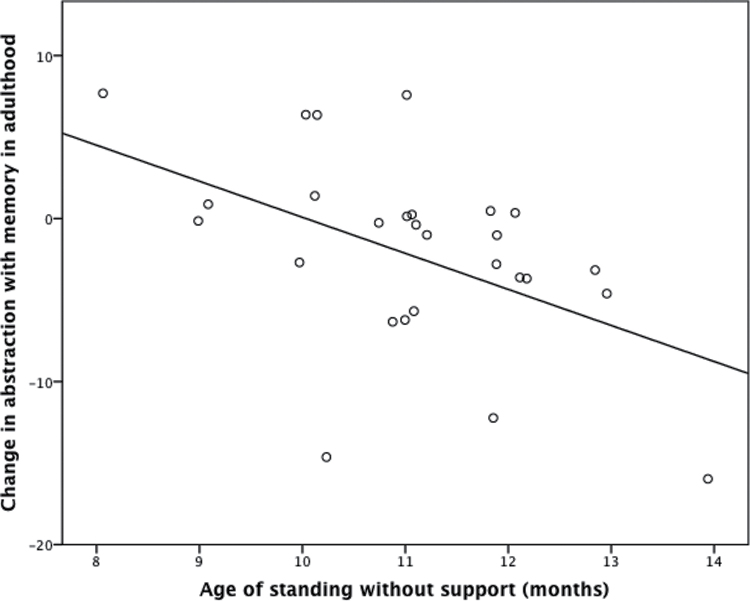
Significant negative correlation between the change in abstraction with memory from age 34 to 43 and age at learning to stand in infancy subjects with schizophrenia. Jitter added to show overlapping points.

Data were available on abstraction with memory and age at learning to stand without support on 27 patients. Bivariate correlation analysis revealed a significant negative correlation between change in abstraction with memory and age of learning to stand without support in schizophrenia patients (*r* = −.511, *P* = .006, figure 1) but not in controls (*r* = .103, *P* = .466). There was no significant association between age of learning to stand and change in mean cognitive function (average of all tests) in schizophrenia (*r* = −.34, *P* = .12) or in controls (*r* = .01, *P* = .97).

Multiple regression revealed that greater decline in abstraction with memory in schizophrenia was significantly predicted by later age of learning to stand without support both without adjustment (beta = 2.2; *t* = 3, *P* = .006), and after adjustment (beta = 2.5; *t* = 2.7, *P* = .01) for gender, exposure to antipsychotic medication, level of function (SOFAS) at both time points, and level of education. A regression model including all subjects with diagnosis, age at learning to stand, and the interaction between age at learning to stand and diagnosis indicated that there was a significant diagnosis by development interaction (beta = 2.5; *t* = 3.4, *P* = .001). This demonstrates that the association between infant motor development and adult cognitive trajectory differs in schizophrenia from the general population.

Finally, we noted that a few patients with schizophrenia scored below chance on the test of abstraction with memory (2 patients at both time points, 1 patient at time 1 only, and 5 patients at time 2 only). Therefore, we repeated our analysis having excluded these “worse than chance” performers. There was still a significant interaction between age at learning to stand and diagnosis on change in abstraction with working memory (beta = 2.0; *t* = 2.5, *P* = .02) and the association within schizophrenia between age at learning to stand and decline in abstraction with memory remained significant (beta = 1.7; *t* = 2.4, *P* = .03)

## Discussion

This is the first report regarding the association between longitudinal change in cognition and a marker of infant neurodevelopment in subjects with schizophrenia. Our results show that the deterioration of abstraction with memory between the ages of 34 and 43 was significantly predicted by the age of the attainment of a neurodevelopmental milestone (learning to stand without support). These findings indicate that subjects with schizophrenia exhibit progressive cognitive dysfunction in adulthood (in at least one domain, though not in all domains) and that this decline may be underpinned by abnormal brain maturation during infancy.

Our finding of progressive decline in executive function in subjects with schizophrenia supports a neurodegenerative model of schizophrenia, whereas the observed association between cognitive decline in adulthood and the age of attainment of a neurodevelopmental milestone (learning to stand without support) argues for the relevance of neurodevelopmental processes in changes that occur much later in life. These findings suggest that neurodevelopmental and neurodegenerative processes in schizophrenia may be mechanistically related. Although our study does not attempt to identify any particular causal agent, plausible candidate processes that could underpin delayed infant neurodevelopment and adult cognitive deterioration include deficits in synaptic plasticity, possibly secondary to genetic factors or to environmental insults in utero or in the perinatal period.

Our finding of abstraction with memory decline is not consistent with those of previous reports of longitudinal change in cognition in schizophrenia. To date, many studies have suggested that cognitive deficits in patients with schizophrenia are stable over time, regardless of symptomatic fluctuations.^34^ A meta-analysis of 53 longitudinal studies found that patients with schizophrenia show significant improvements in most cognitive tasks,^35^ although they may not improve with time as much as controls.^36^ However, previous studies tend not to focus on general population samples and usually do not follow up patients over periods as long as the 9-year follow-up employed in our study. As far as we know, this is the first birth cohort study to examine adult cognitive change in schizophrenia, and the fact that all the participants were the same age, eliminating any confounding from this factor, may have also contributed to the sensitivity of our experimental design.

Previous longitudinal studies have used different cognitive tasks to that used in our study, and it is possible that the AIM measure of abstraction with memory could be especially sensitive to cognitive decline. Accumulating evidence suggests that the volume of some brain regions relevant to executive/working memory function decreases progressively over the course of schizophrenia,^17,18^ which one would plausibly expect to be accompanied by deterioration in function. We have previously shown that performance in the AIM test is associated both with precocity of infant motor development and adult brain structure in the general population,^7^ and thus it appears sensitive to the underlying biology relevant to brain development and degeneration in schizophrenia. As our cognitive measures were few in number, it is hard for us to make very firm conclusions about the specificity of the observed effect to one domain of cognition. Thus, although we can infer that a measure of cognitive decline is linked to neurodevelopment in schizophrenia, further studies will be required to examine how generalizable these results are to other cognitive functions.

Some methodological limitations of our study should be considered. The modest number of subjects in the current study may limit the generalizability of the findings. Nevertheless, the birth cohort design, with all subjects being the same age, helps to minimize variance according to age variation, and the population-based sampling helps ensure the generalizability, compensating to an extent for the modest sample size. The level of detail of our developmental measure and cognitive measures is crude in comparison to the level of detail of assessment employed in many cross-sectional studies of child development or of adult neuropsychological function. We emphasize that the developmental delay we observe in our study in schizophrenia is subtle, and not at the level that would be viewed as clinically significant by a pediatrician. We use precocity of learning to stand unsupported as a crude continuous measure of neurodevelopmental integrity rather than focus on the small number of patients with schizophrenia who have severe developmental delay. We acknowledge that a limitation of our study is the lack of a full-scale IQ assessment. However, the novelty of our study is that it combines infancy and adult longitudinal data, and our very long follow-up, from data collected in infancy to the fifth decade of life, means that these data are unique and can make a valuable contribution to understanding the course of schizophrenia over the life span. As is to be expected, there was significant sample attrition over a long follow-up study, but our attrition analysis indicates that study completers are representative of study starters in many respects. Our population-based design means that we can also quantify in some respects how representative our study completers are of all the patients in cohort of schizophrenia, even including information about patients in the birth cohort who had died before this study began or who declined to take part in the study.

## Conclusions

Our prospective study showed that a prospectively collected marker of infant neurodevelopment predicts deterioration of a measure of executive function in schizophrenia. These findings suggest that developmental and degenerative aspects of schizophrenia may be 2 manifestations of a common underlying process.

## Funding

The study was supported by the UK Medical Research Council (G0701911), the Academy of Finland, the Sigrid Juselius Foundation, the Stanley Foundation, and the Brain and Behavior Research Fund, and was conducted in part within the University of Cambridge Behavioural and Clinical Neuroscience Institute, supported by a joint award by the Medical Research Council and Wellcome Trust (G1000183 and 093875/Z/10/Z). The funding bodies did not participate in the design and conduct of the study; collection, management, analysis, and interpretation of the data; or the preparation, review, or approval of the manuscript.
